# P071: When rehabilitation and reeducation rhyme with infection and prevention

**DOI:** 10.1186/2047-2994-2-S1-P71

**Published:** 2013-06-20

**Authors:** J Sztajzel, D Pittet, B Huttner

**Affiliations:** 1 Infection Control Programme, HCUGE, Genève 4, Switzerland

## Introduction

The infection control measures implemented for patients carrying multidrug resistant organisms (MDRO) often interfere drastically with the arsenal of rehabilitation therapies. In rehabilitation wards these therapies constitute the main reason for hospitalization, thus creating an apparent conflict between infection prevention and rehabilitation.

## Objectives

The objective of this study was to evaluate the implementation of an itemized document template that allows the adaptation of specific infection control measures to the particular situation of each MDRO carrier.

## Methods

We implemented a document for the infection control management of carriers of vancomycin-resistant enterococci, multidrug-resistant *Acinetobacter baumannii* and carbapenem resistant Enterobacteriaceae in an academic rehabilitation hospital with 193 beds in Geneva, Switzerland. The document specifies individualized restrictions regarding the movements of patients within the hospital with the aim to make it possible for the patient to participate in re-education and rehabilitation activities that take place outside the patient room as much as possible (e.g. a patient may be allowed to go to physiotherapy room, while he may not use the hospital swimming pool). The document is adapted each week based on the clinical evolution of the patient and after discussion with the nursing team. In order to assess the perceived utility of the document we conducted a structured survey with head nurses (HN) and infection control nurses (ICN).

## Results

During the period from October 2011 to February 2013 the document was used in 9 patients. In March 2013 the survey was sent to 8 HN and 9 ICN. The overall response rate was 82% (14/17). All respondents judged the document to be often useful and to be a frequent reference source, 3/14 ( HN only) thought that it sometimes could be better adapted to the individual patient, yet its level of detail was judged as good by all participants. Furthermore, 7/14 ( HN and ICN ) respondents suggested slight modifications to the document.

## Conclusion

Our experience and the results of the survey indicate that an individualized document allows patients carrying MDRO to better participate in rehabilitation activities despite the implementation of adequate infection control measures.

## Disclosure of interest

None declared

